# Recovery of Motor Imagery Ability in Stroke Patients

**DOI:** 10.1155/2011/283840

**Published:** 2011-04-05

**Authors:** Sjoerd de Vries, Marga Tepper, Bert Otten, Theo Mulder

**Affiliations:** ^1^Centre for Human Movement Sciences, University Medical Centre Groningen, University of Groningen, P.O. Box 196, 9700 AD Groningen, The Netherlands; ^2^Centre for Rehabilitation, University Medical Centre Groningen, 9700 RD Groningen, The Netherlands; ^3^Royal Netherlands Academy of Arts and Sciences, 1011 JV Amsterdam, The Netherlands

## Abstract

*Objective*. To investigate whether motor imagery ability recovers in stroke patients and to see what the relationship is between different types of imagery and motor functioning after stroke. *Methods*. 12 unilateral stroke patients were measured at 3 and 6 weeks poststroke on 3 mental imagery tasks. Arm-hand function was evaluated using the Utrecht Arm-Hand task and the Brunnström Fugl-Meyer Scale. Age-matched healthy individuals (*N* = 10) were included as controls. *Results*. Implicit motor imagery ability and visual motor imagery ability improved significantly at 6 weeks compared to 3 weeks poststroke. *Conclusion*. Our study shows that motor imagery can recover in the first weeks after stroke. This indicates that a group of patients who might not be initially selected for mental practice can, still later in the rehabilitation process, participate in mental practice programs. Moreover, our study shows that mental imagery modalities can be differently affected in individual patients and over time.

## 1. Introduction

Recently, several researchers have proposed the use of mental practice to facilitate motor recovery in stroke patients and other patients with motor disorders [1, 2]. Mental practice is a training method where imagination of movements, without actually moving, is used with the intention of improving motor performance. In other words, mental practice is the imagined rehearsal of a movement with the specific intent of improving that movement [3]. Several lines of research have shown that mental practice improves motor performance in healthy participants [4, 5] as well as in stroke patients [6–9]. Mental practice is suggested to work because the motor control structures in the brain are activated in more or less the same way as during the actual performance of movements [10, 11].

Indeed, studies with healthy individuals have shown that motor imagery and actual action share some striking similarities. When someone is asked to perform a movement, for example, “walk along this line”, and to imagine the same movement, the time to complete the actual walking movement is similar to the time needed for completing the imagined walking movement [12]. Moreover, neuroimaging studies have shown that during motor imagery the same brain areas are active as during actual movement [13–17]. Hence, also in stroke patients there should be a relation between motor function and motor imagery ability. However, the reported results thus far are not consistent [18].

One factor that might explain these ambiguous results, as suggested by Daprati et al. [18], is the heterogeneous manner in which motor imagery ability is assessed. The researchers that have used motor imagery assessments have used different types of motor imagery tasks. Motor imagery ability is a complex cognitive capacity which is, until now, not fully understood and has an intricate relationship with other types of imagery. First, motor imagery can be divided into two different types. We can explicitly imagine movements of a limb. Here imagery is conscious and involves, for example, the voluntary active imagination of “reaching for a cup on the table with your right arm”. This type of imagery is known as explicit motor imagery and there exist a number of studies with stroke patients that used imagery tasks that depended on explicit motor imagery [19–25]. On the other hand, we can be tricked into motor imagery implicitly by, for example, answering a question about the handedness (left-right) of a picture of a hand, or by answering with which kind of grip we would prefer to grasp a cup of coffee or a dowel in a particular orientation. This type of imagery is known as implicit motor imagery and is also used in a number of studies with stroke patients [26–29]. 

Secondly, motor imagery is also related to other cognitive processes such as reflected in tasks that rely on the mental rotation of pictures, so called visual imagery tasks. A well-known example of visual imagery is the Shepard Meltzer task where two three-dimensional figures are pictured side to side in different orientations, and the respondent has to answer the question whether these figures are similar or not [30]. Recent neuroimaging data have shown that, although these different types of imagery, implicit, explicit and visual imagery, share similar neural processes, at the same time they also differ in the underlying mechanisms [31–34] suggesting that these types of imagery can be affected differently after hemiparetic stroke. 

A second factor that could explain why the relationship between motor function and motor imagery is less clear after stroke, is related to the poststroke moment when motor imagery ability is assessed. The differences in the time since stroke when motor imagery was assessed varies largely between studies, ranging from weeks [28] to years [29] poststroke. Stroke has, indeed, more cognitive effects early after stroke than later after stroke [35] therefore, performance on motor imagery ability assessment could well be influenced by the moment of assessment. In other words, the moment of assessment could differently affect the imagery modalities that are used in the experiments of individual stroke patients.

Until now, no study has followed the recovery process of motor imagery and motor functioning, in parallel during the rehabilitation period. Because of the multifaceted nature of motor imagery and its relation to visual imagery the present study was designed to assess the relation of different imagery types and motor function to see to what extend they relate and if they recover in parallel after 3 weeks. The purposes of the research was (1) to measure imagery ability of hemiparetic stroke patients 3 weeks poststroke and 6 weeks poststroke (2) to find out whether motor imagery ability improved in parallel to arm-hand functioning and (3) to see what relationship exists between different types of imagery and motor functioning after hemiparetic stroke.

## 2. Methods

### 2.1. Subjects

Twelve hemiparetic stroke patients (4 female, mean  age  =  59.75 years, SD  =  11.98 years, 1 left handed) who suffered a first unilateral stroke 3 weeks earlier (*M*  =  22.8 days; SD  =  3.5 days) participated in this study. The patients were recruited from the stroke unit of a rehabilitation centre. Six patients were classified as left hemiparetic and 6 patients were classified as right hemiparetic. All subjects gave informed, written consent. The experiment was conducted in accordance with the Declaration of Helsinki and approved by the local ethics committee of the medical centre of the University of Groningen.

The inclusion criteria were patients with a hemiparetic arm/hand secondary to a stroke, with no explicit age limit. Exclusion criteria were multiple strokes, comorbidity which interfered with the objectives of the study, severe perceptual problems and severe cognitive impairments, severe aphasia, and other neurologic conditions that interfered with the goal of the study. During the study, the rehabilitation program remained unaltered for all participating patients. Age-matched healthy individuals (*N* = 10,5 female, mean age 55, SD  =  10.22 years, 2 left handed) were included as a control group.

### 2.2. Instruments

#### 2.2.1. Implicit Motor Imagery Ability

Motor imagery ability was measured by means of a mental rotation task, namely, a hand laterality judgement task, known for its clinical value in measuring motor imagery ability [36–38]. Subjects had to decide as fast as possible whether a rotated picture of a hand on a computer screen was a left or a right hand by pushing 1 of 2 buttons (L/R) with their nonimpaired limb.

#### 2.2.2. Visual Imagery

A mental rotation task known to depend on a visual imagery strategy was used to measure visual imagery [34]. In the visual imagery task, subjects were asked to determine as fast as possible whether a rotated picture was a normal canonical letter or its mirror image by pushing 1 of 2 buttons with their nonimpaired hand. 

The order of the implicit motor imagery task and the visual imagery task was counterbalanced between subjects. The implicit motor imagery and visual imagery tasks were both divided in 4 blocks. Each block contained 72 stimuli, yielding a total of 288 stimuli per task. Prior to each task, a practice block was presented, containing 48 stimuli. All stimuli were presented randomly at the centre of the computer screen with a random delay between 2 and 3 s. The stimuli were rotated, presented in 60° increments from the upright position between 0° and 300°. Zero degrees for hand stimuli was a hand pictured in an upright position with fingers pointing upwards. Zero degrees for letter stimuli was a picture of the letter F or R in an upright position. Stimuli remained on the screen until the participant responded, or when 10 s had expired. After each block, there was a short rest period. For each task the mean accuracy scores (ACC) for each subject were calculated as the proportion of correct responses, yielding a minimum score of 0% (all wrong) and a maximum score of 100% (all correct). For each task the mean reaction times (RT) for each subject were calculated on RTs between 350 ms and 10,000 ms (excluding anticipated responses).

In both mental imagery tasks, responses were given by pushing 1 of 2 buttons with the nonparetic hand. This hand could be either the dominant or the nondominant hand. To control for confounding dominant nondominant hand effects, the control group executed the mental imagery task with both the dominant and nondominant hand in a counterbalanced order. For the rest of the experiment, all conditions for patients and controls were equal.

#### 2.2.3. Explicit Motor Imagery Ability

**Figure 1:**
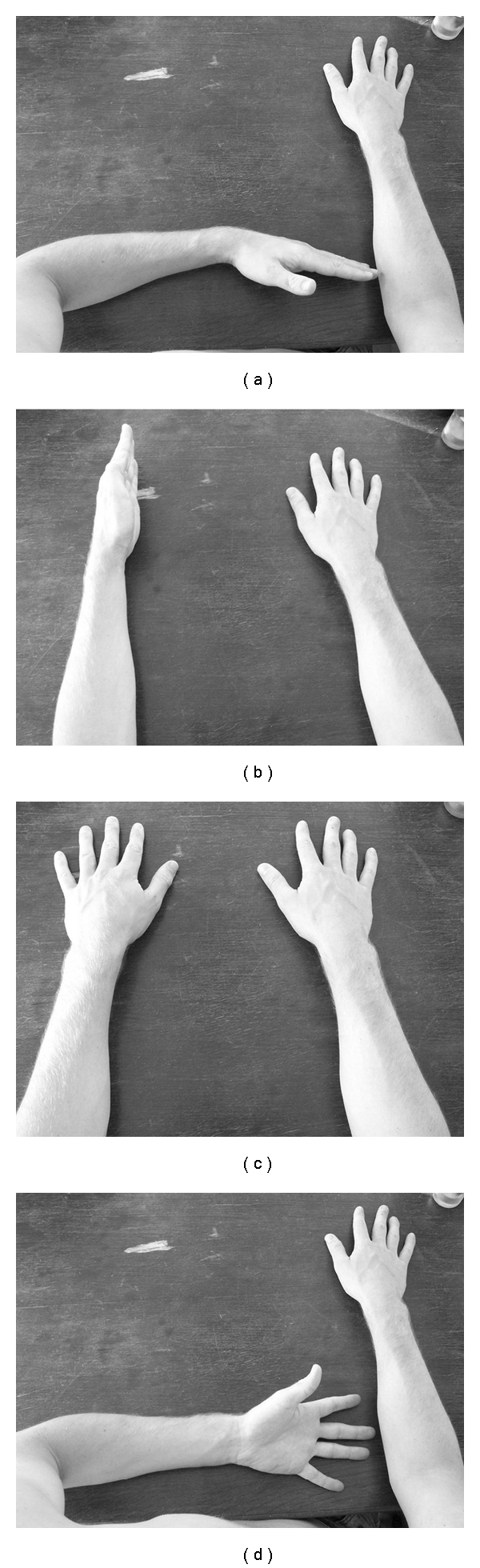
Example of a 4-choice solution of the arm end position after the instructions in the explicit motor imagery task.

The hand laterality task measured motor imagery implicitly. However, since implicit and explicit motor imagery measure different components of motor imagery, we also used a motor imagery task that measured the explicit motor imagery ability of the patients. During explicit imagery tasks, subjects were asked to imagine moving their limbs in a particular way (e.g., “imagine flexing your underarm 90 degrees”). We asked subjects to close their eyes and to imagine as vividly as possible a movement with their hand, without actual movement, in a series of steps. For example, stepwise instruction for one item of the explicit motor imagery task were “Step 1: Place both hands, palm facing down on the marks on the table in front of you. Step 2: Turn your wrist 90 degrees until your palm is facing inwards. Step 3: Flex your underarm 90 degrees until it your hand is touching your chest. Step 4: Flex your arm upwards 90 degrees until your hand is pointing to the ceiling. Step 5: Extend your arm 90 degrees until it reaches the tabletop”. After the instructions, the subjects had to indicate what the position of his or her imagined hand was, by choosing the correct hand position from 4 pictured examples of arm-hand configurations (see Figure 1). The 4-choice solution contained one correct end position. All other answers were false. The researcher compared the answer given by the participant with the accurate hand position. The explicit motor imagery task consisted of 12 items (6 for the paretic arm and 6 for the nonparetic arm). If all answers were correct, a total maximum score of 12 could be reached. 

#### 2.2.4. Utrechtse Arm-Hand Task (UAT)

Hand function was measured with the Utrecht Arm-Hand task (UAT) [39]. The UAT consists of a hierarchical ordinal scale, ranging from 0 (nonfunctional arm) to 7 (clumsy arm).

#### 2.2.5. Brunnström Fugl-Meyer Scale (BFM)

The functional level of the patients was measured with the Brunnström Fugl-Meyer Scale (BFM). This test measures motor recovery in patients with hemiplegia following stroke [40]. The BFM scale includes items related to movements of the shoulder, elbow, forearm, wrist, and hand in the upper extremity, as well as the hip, knee, and ankle in the lower extremity. For the present study, only the upper extremity was measured. Each item was scored on a 3-point ordinal scale (0: cannot perform, 1: performs partially, 2: performs fully). The total score on the upper extremity part of the BFM ranged from 0 (hemiplegia) to a maximum of 66 points (normal motor performance).

### 2.3. Procedure

To measure the recovery of motor imagery ability and arm-hand function, each patient was assessed 2 times: 3 weeks poststroke and 6 weeks poststroke. During the measurements, all subjects sat at a table in a chair with a backrest. Subjects were instructed to refrain from any actual movements during motor imagery tasks. In between the two measurement sessions the patients continued with their regular treatment program. The control group, who acted as a reference value to determine normal motor imagery ability, was measured once during the research period.

### 2.4. Statistical Analyses

Group differences between patients and controls on implicit motor (ACC and RT), visual (ACC and RT), and explicit motor imagery 3 weeks poststroke, were determined by a Mann-Whitney *U* Test with exact *P* values. Subsequently, a modified *t*-test [41] with *P* < .05 was used to compare individual patients' scores with the control group to determine individual impairment of motor imagery. The correlation between mental imagery measures and motor function measures in the patients group was determined by calculating Pearson correlations between all measures on 3 weeks poststroke and by calculating Pearson correlations between all measures on 6 weeks poststroke. To determine whether mental imagery ability and motor function improved at 6 weeks poststroke compared to 3 weeks poststroke, a Wilcoxon Signed-Ranks Test on implicit motor imagery (ACC and RT), visual imagery (ACC and RT), explicit motor imagery, UAT, and BFM was used. All data were analyzed with SPSS version 16.0 (SPSS, Inc., Chicago, IL, USA).

## 3. Results

### 3.1. Three Weeks Poststroke

#### 3.1.1. Imagery Ability: Patients versus Controls

**Figure 2:**
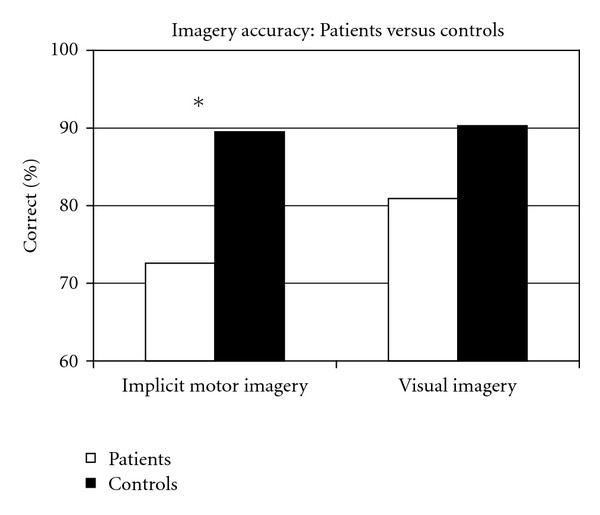
Differences between Patients (white bars) and Controls (black bars) on mean implicit motor and visual imagery accuracy. Asterisk indicates significant differences. Patients were less accurate then controls on implicit motor imagery at 3 weeks poststroke (*P* = .003). The difference between patients and controls on mean visual imagery accuracy was not significant (*P* = .136).

Figure 2 shows the mean accuracy scores of the control group compared with the patients group for the implicit motor and the visual imagery task. The Mann-Whitney *U* Test showed that the patients had a significantly lower implicit motor imagery accuracy score then controls (*U* = 16.5, *P* = .003). No significant differences existed between the patients and the control group on the visual imagery accuracy score (*U* = 37, *P* = .136) and the explicit motor imagery task (*U* = 46.5, *P* = .382). The reaction times for the implicit motor imagery task and the visual imagery task did not differ significantly (with *U* = 48, *P* = .456, and *U* = 46, *P* = .381, resp.). 

#### 3.1.2. Imagery Ability: Individual Differences

Comparison of the individual patients' scores with the control group mean with a modified *t*-test with (*P* < .05) showed that one patient had a lower score on visual imagery selectively. Four of the 12 patients scored significantly below mean accuracy of the control group on the implicit motor imagery task. From these 4 patients that were impaired on the implicit motor imagery task, 2 patients also differed significantly from the control group on the visual imagery task and the other 2 patients were selectively impaired on the implicit motor imagery task. Also, 2 patients differed significantly from the control group on the explicit motor imagery task. Patient 9 scored significantly below the mean accuracy of the control group on the implicit motor imagery task and the explicit motor imagery task. Patient 7 scored significantly below the mean accuracy of the control group on all 3 imagery tasks (see Table 1). These results show that 33% of the patients in this study had impaired motor imagery ability and 67% had unimpaired motor imagery.

#### 3.1.3. Correlations between Mental Imagery and Motor Function Measures at 3 Weeks Poststroke

Table 2 shows the correlations between mental imagery and motor function measures. There is a high significant positive correlation between the motor function measures UAT and BFM while the correlations between the mental imagery measures of implicit motor imagery, visual imagery and explicit motor imagery, and the UAT and BFM are low to moderate and not significant. Furthermore, Table 2 shows a significant strong positive correlation of implicit motor imagery accuracy with visual imagery accuracy and a significant high positive correlation with explicit motor imagery. 

### 3.2. Six Weeks Poststroke

#### 3.2.1. Recovery of Imagery Ability and Motor Function

**Figure 3:**
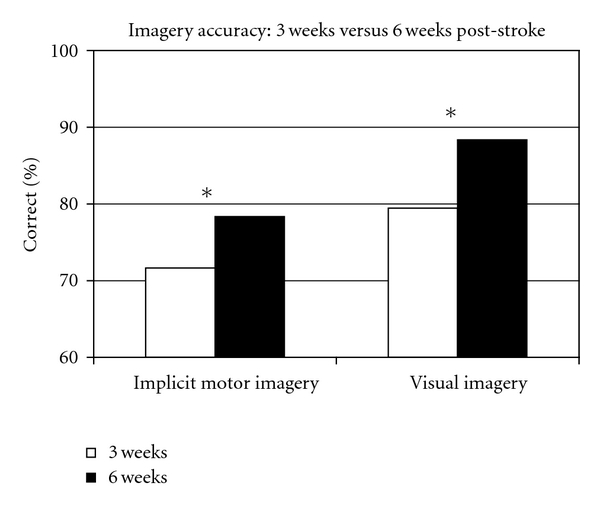
Difference between 3 weeks (white bars) and 6 weeks (black bars) patients poststroke mean implicit motor and visual imagery accuracy scores. Asterisk indicates significant differences. Patients were more accurate on implicit motor imagery and visual imagery at 6 weeks poststroke compared to 3 weeks poststroke (*P* < .01).

One patient dropped out after the 3 weeks poststroke measurements. At 6 weeks poststroke the 11 remaining patients were more accurate on the implicit motor imagery and visual imagery tasks compared to 3 weeks poststroke (*z* = −2.76, *P* = .006 and *z* = −2.81, *P* = .005) (see Figure 3). Reaction time on the implicit motor imagery and the visual imagery tasks was not different at 6 weeks compared to 3 weeks poststroke (*z* = −1.16, *P* = .248 and *z* = −1.07, *P* = .286). The performance on explicit motor imagery was also not different at 6 weeks compared to 3 weeks poststroke (*z* = −.54, *P* = .591). 

Motor function did improve over 3 weeks. Patients performed marginally significantly better on UAT at 6 weeks poststroke compared to 3 weeks poststroke (*z* = −1.84, *P* = .066). And patients performed significantly better on BFM at 6 weeks poststroke compared to 3 weeks poststroke (*z* = −2.68, *P* = .007) (see Table 3).

#### 3.2.2. Recovery of Motor Imagery Ability: Individual Differences

Of the 4 patients that had impaired motor imagery at 3 week poststroke, in 2 patients recovery of motor imagery ability was seen at 6 weeks poststroke. Patient 3 and patient 7 were both more accurate on implicit motor imagery 6 weeks poststroke, whereas patient 2 and 9 showed no increase in accuracy. Their implicit motor imagery level remained around chance level at 6 weeks poststroke.

#### 3.2.3. Correlations between Mental Imagery and Motor Function Measures at 6 Weeks Poststroke

Table 5 shows the correlations between mental imagery and motor function measures at 6 weeks poststroke. There is a highly significant positive correlation between the motor function measures UAT and BFM. The correlations between the mental imagery measures and the UAT and BFM are low to moderate and are not significant. Table 5 also shows a significant high positive correlation between implicit motor imagery and explicit motor imagery. The significant strong positive correlation of implicit motor imagery with visual imagery at 3 weeks poststroke is weaker and no longer significant at 6 weeks poststroke (Table 4).

## 4. Discussion

In the present study, we attempted to answer the question whether motor imagery ability recovers in hemiparetic stroke patients. Our results show that implicit motor imagery ability, indeed, improved significantly after 3 weeks. Moreover, 2 patients with impaired motor imagery ability also improved from performing motor imagery at around chance level at 3 weeks poststroke to motor imagery ability above chance level at 6 weeks poststroke. When patients are not able to imagine movements of their affected limb, it is useless to confront them with mental practice exercises during their rehabilitation program [42]. It is, therefore, important to measure motor imagery ability of stroke patients before including them in motor imagery training. Indeed, several researchers have used motor imagery ability tests as a screening tool before including patients to mental practice training programs [19–27]. Previous research has suggested that motor imagery capacity could diminish with time poststroke, that is, patients more then a year poststroke were less accurate on motor imagery tasks than patients a few weeks after stroke [28, 29]. Also, in a recent study by Daprati et al. [18], response times were longer and accuracy scores were lower in chronic stroke patients compared to patients who ranged between 6 and 20 weeks poststroke on a motor imagery task involving grip selection. These results suggest that mental practice could be most effectively introduced in the rehabilitation process early after stroke. However, our findings show that early inclusion in a mental practice program could still be problematic. Indeed, the results show that when a motor imagery screening is used in the first weeks after stroke, a percentage of the patients would be excluded although they might have the potential to benefit from mental practice later in their recovery progress. 

One explanation for the difference between 3 weeks and 6 weeks poststroke might be found in the relation of motor imagery with other types of mental imagery. The patients in our study also performed better on visual imagery at 6 weeks poststroke, compared to 3 weeks poststroke, showing that recovery of imagery was not exclusive for implicit motor imagery. We found a significant correlation between visual and implicit motor imagery at 3 weeks poststroke. However, this correlation was no longer significant at 6 weeks poststroke. A high correlation existed between implicit and explicit motor imagery, 3 weeks as well as 6 weeks poststroke. The fact that implicit motor imagery and visual imagery were strongly correlated early after stroke might suggest that early after stroke both tasks rely on similar mechanism. Kosslyn and colleagues showed that the use of a visual or motor imagery strategy to solve a mental rotation task can be voluntarily adopted [43]. It might be that when patients are not able to use a motor imagery strategy to solve motor imagery tasks, they refer to a more visual-spatial strategy involving visual imagery. These results are in accordance with the Daprati et al. [18] study, who also suggested that in some patients a more visual strategy can be used to solve motor imagery problems. Although these correlations are based on a study with a small sample size, they indicate that in the first 6 weeks poststroke the different imagery modalities might be differently affected and furthermore, they suggest that visual imagery ability is a profound factor during testing of motor imagery ability early poststroke and that the role of this factor diminishes over time. 

Our results regarding the relationship between motor function and motor imagery measures also seem to suggest this. Although motor function did improve significantly after 3 weeks, the correlation between measures of motor function and mental imagery measures were low and not significant on 3 weeks poststroke and even lower on 6 weeks poststroke, indicating independent recovery of motor imagery and motor function early after stroke. Taken together, these results suggest that early after stroke, motor imagery ability is more related to visual imagery capacity than to actual motor function. It seems plausible, however, that across time the relationship between motor function and motor imagery becomes stronger as a result of a more frequent use of intact motor imagery to solve the motor imagery tasks. A long term follow-up study could determine if and how this relation changes over time. 

These results are relevant for rehabilitation practice. Recent research shows that motor imagery is associated with activation in a cortico-subcortical network related to the planning and execution of movements, involving frontoparietal regions and the basal ganglia (see [44] for a review). Visual imagery is thought to be associated with the activation of a parieto-occipital network supporting visual spatial functions [34]. The advantage of mental practice is thought to be greatest with motor imagery because this type of imagery involves activation of the same neural structures that are involved in planning and execution of actions. Our study did not focus on specific lesion locations of individual patients. However, previous research has shown that damage to the parietal cortex and basal ganglia affects motor imagery ability [45, 46]. On the other hand, patients have been reported with lesions of the parietal cortex showing intact motor imagery ability [28, 29]. Also, a recent study has reported patients with subcortical stroke that affected motor imagery ability and the same study also reported unimpaired motor imagery performance after subcortical stroke [47]. Moreover, the patients with intact motor imagery ability showed activation of cortical motor areas during motor imagery, demonstrating the potential for motor imagery to target the motor system in subcortical stroke patients. These results are promising and advocate that motor imagery can still be used for rehabilitation despite lesions of the motor system. Therefore, screening solely based on lesion location seems an insufficient method for patient inclusion. If patients use different strategies for solving motor imagery tasks over time, for example, if there are patients that use more visual strategies and patients that use more motor-based strategies depending on where they are in their recovery process, it would be expected that a different neural architecture is involved in these strategies and that involvement of this architecture could also change over time. Our results suggest that individual patients are able to change their strategies across time. Therefore, they might benefit from different mental practice instructions depending on which modality can be used in their recovery process. However, the exact involvement of cortico-subcortical networks supporting these different imagery modalities and its change across time is unclear and should be addressed in future research.

## 5. Conclusions

The results of the present study showed that motor imagery recovers after stroke. These findings indicate that if motor imagery screening takes place during the first weeks after stroke, a substantial group of patients, who showed impaired motor imagery in the first weeks after stroke, would be scored as unimpaired later on, which means that a group of patients who would not be selected early after stoke can still participate in a mental practice rehabilitation program later in the rehabilitation process. Moreover, we showed that the extent of the impairment of the different types of mental imagery varies in individual patients and across time, with a particular involvement of visual imagery ability at 3 weeks poststroke compared to 6 weeks poststroke, showing that motor imagery ability is a complex capacity, related to other forms of mental processes. This makes reliable screening of stroke patients a delicate task. More follow-up research is needed to gain insight into the recovery of and relation between mental imagery strategies and motor function.

## Figures and Tables

**Table 1:** Mean accuracy scores for patients that were impaired on the implicit motor imagery and, or the visual imagery task and, or the explicit motor imagery task versus control subjects (SD in parentheses) at 3 weeks poststroke. Impairment of individual patients versus controls was tested with a modified *t*-test, with *P* < .05. Table shows that 7 of the 12 patients were not impaired on any of the imagery tasks. One patient was impaired on the visual imagery task selectively, 2 patients were impaired on implicit motor and visual imagery simultaneously, and 2 patients were impaired on implicit motor imagery selectively.

	Implicit motor imagery	Visual imagery	Explicit motor imagery	
Controls	89.5 (6.8)	90.3 (10.6)	7.5 (2.0)	

Patients				

*No impairment of imagery*				
1	96	99	10	
4	81	95	8	
5	85	94	6	
6	83	85	7	
8	84	90	8	
10	77	73	7	
11	83	97	8	
*Selective impairment of visual imagery*				
12	73	57	6	
*Impairment of motor imagery*				
*Selectively*				
3	47	70	6	
9	53	93	0	
*Simultaneous with visual imagery*				
2	52	56	6	
7	57	62	0	

**Table 2:** Correlations between mental imagery and motor function measures of patients at 3 weeks poststroke.

	UAT	BFM	ACC implicit motor imagery	RT implicit motor imagery	ACC visual imagery	RT visual imagery	Explicit motor imagery
UAT	1	0.96***	−0.18	0.14	0.18	0.14	−0.44
BFM		1	−0.26	0.19	0.12	0.15	−0.38
ACC implicit motor imagery			1	0.24	0.63*	−0.13	0.71**
RT implicit motor imagery				1	0.46	0.56	0.05
ACC visual imagery					1	−0.01	0.34
RT visual imagery						1	−0.55
Explicit motor imagery							1

*P < .05,**P = .01,***P < .001.

**Table 3:** Mean motor function scores and SD of patients at 3 weeks and 6 weeks poststroke. Patients performed marginally significantly better on UAT (*P* = .066) and significantly better on BFM (*P* = .007) at 6 weeks poststroke compared to 3 weeks poststroke.

Motor function	3 weeks (*n* = 11)	6 weeks (*n* = 11)	
	Mean (SD)	Mean (SD)	Difference score
UAT	4.6 (2.5)	5.3 (2.3)	0.7
BFM	42.5 (21.6)	48.5 (19.4)	6.0

**Table 4:** Mean accuracy scores for patients that were impaired on implicit motor imagery at 3 weeks poststroke (*n* = 4). Table shows recovery of implicit motor imagery ability between 3 and 6 weeks poststroke in 2 patients with impaired implicit motor imagery ability.

	Implicit motor imagery (3 weeks)	Implicit motor imagery (6 weeks)	Visual imagery (3 weeks)	Visual imagery (6 weeks)	Explicit motor imagery (3 weeks)	Explicit motor imagery (6 weeks)	
Patients							

*No recovery of motor imagery*							
2	52	54	56	68	6	5	
9	53	54	93	94	0	0	
*Recovery of motor imagery*							
3	47	61	70	93	6	3	
7	57	74	62	68	0	4	

**Table 5:** Correlations between mental imagery and motor function measures of patients 6 weeks poststroke.

	UAT	BFM	ACC implicit motor imagery	RT implicit motor imagery	ACC visual imagery	RT visual imagery	Explicit motor imagery
UAT	1	0.98**	−0.32	0.00	−0.04	−0.11	−0.37
BFM		1	−0.32	0.01	−0.06	−0.15	−0.27
ACC implicit motor imagery			1	0.37	0.47	−0.10	0.71*
RT implicit motor imagery				1	0.41	0.73*	−0.04
ACC visual imagery					1	−0.03	0.37
RT visual imagery						1	−0.52
Explicit motor imagery							1

*P < .05, **P < .001.
